# Epigenetic Modifications of the PGC-1α Promoter during Exercise Induced Expression in Mice

**DOI:** 10.1371/journal.pone.0129647

**Published:** 2015-06-08

**Authors:** Timothy L. Lochmann, Ravindar R. Thomas, James P. Bennett, Shirley M. Taylor

**Affiliations:** 1 Massey Cancer Center, Virginia Commonwealth University, Richmond, Virginia, United States of America; 2 Parkinson’s Center, Virginia Commonwealth University, Richmond, Virginia, United States of America; 3 Department of Neurology, Virginia Commonwealth University, Richmond, Virginia, United States of America; 4 Department of Psychiatry, Virginia Commonwealth University, Richmond, Virginia, United States of America; 5 Department of Physiology and Biophysics, Virginia Commonwealth University, Richmond, Virginia, United States of America; 6 Department of Microbiology and Immunology, Virginia Commonwealth University, Richmond, Virginia, United States of America; University of Wisconsin, UNITED STATES

## Abstract

The transcriptional coactivator, PGC-1α, is known for its role in mitochondrial biogenesis. Although originally thought to exist as a single protein isoform, recent studies have identified additional promoters which produce multiple mRNA transcripts. One of these promoters (promoter B), approximately 13.7kb upstream of the canonical PGC-1α promoter (promoter A), yields alternative transcripts present at levels much lower than the canonical PGC-1α mRNA transcript. In skeletal muscle, exercise resulted in a substantial, rapid increase of mRNA of these alternative PGC-1α transcripts. Although the β_2_-adrenergic receptor was identified as a signaling pathway that activates transcription from PGC-1α promoter B, it is not yet known what molecular changes occur to facilitate PGC-1α promoter B activation following exercise. We sought to determine whether epigenetic modifications were involved in this exercise response in mouse skeletal muscle. We found that DNA hydroxymethylation correlated to increased basal mRNA levels from PGC-1α promoter A, but that DNA methylation appeared to play no role in the exercise-induced activation of PGC-1α promoter B. The level of the activating histone mark H3K4me3 increased with exercise 2–4 fold across PGC-1α promoter B, but remained unaltered past the canonical PGC-1α transcriptional start site. Together, these data show that epigenetic modifications partially explain exercise-induced changes in the skeletal muscle mRNA levels of PGC-1α isoforms.

## Introduction

The transcriptional coactivator, PGC-1α, has been linked to mitochondrial biogenesis [1–4]. The close interaction between PGC-1α and mitochondrial transcription factors [3], such as the nuclear respiratory factor (NRF) family, has led to PGC-1α being coined the “master regulator of mitochondria” [4]. In addition to mitochondrial biogenesis, PGC-1α is involved in the regulation of fatty acid oxidation, glucose metabolism, and antioxidants [5–7]. However, recent studies have found that the PGC-1α gene contains more than one promoter and transcribes multiple isoforms [8–14].

A second promoter (promoter B) of PGC-1α has been identified in mice, located approximately 13.7kb upstream of the canonical promoter (promoter A) [9]. This alternate promoter B drives transcription of an alternate exon 1 with two splice donors, generating two new N-termini of PGC-1α. Furthermore, alternative splicing from promoter B generates at least three different isoforms [10, 14]. Microarray studies indicate that these newly identified isoforms activate different genes than the canonical PGC-1α, including genes involved in muscle hypertrophy [10]. While overexpression of canonical PGC-1α strongly activated genes involved in mitochondrial biogenesis and function, overexpression of a protein expressed from promoter B weakly increased mitochondrial genes, and instead activated genes involved in cellular growth pathways. Additionally, a truncated form of PGC-1α expressed from promoter A named NT-PGC-1α, was found to have a similar role to the canonical PGC-1α protein [14]. That this isoform was similar to the promoter B isoform related to hypertrophy [10], with the first exon being the difference, suggests that the genes controlled by PGC-1α are strongly influenced by the N-terminus of the protein.

An analogous promoter has also been identified in human tissues, which is also activated upon exercise [10, 13, 15, 16]. Furthermore, in human brain tissue, eight new isoforms were identified, with putative transcription start sites ~500kb upstream of the canonical promoter [11]. However, the promoters regulating transcription of these new isoforms have not yet been located, and it is not yet known whether any of these alternate mRNA transcripts produce functional protein. Together, these data suggest a much more complex role for the PGC-1α gene than originally thought.

The activation of PGC-1α within different tissues responds to different stressors. Upon exposure to cold, the brown adipose tissue of mice exhibits a marked increase in mRNA transcripts generated from PGC-1α promoter B [8, 15, 17]. Similarly, skeletal muscle tissue from exercised mice yields a marked increase in the levels of mRNA produced from promoter B, whereas mRNA derived from promoter A remains relatively unchanged [8, 9, 12]. The level of total PGC-1α mRNA positively correlates with exercise intensity [12]. Administering clenbuterol to mice causes an increase in mRNA transcripts driven from promoter B of PGC-1α without exercise, indicating that mRNA increases via the β_2_-adrenergic pathway [8, 12]. Human promoter B PGC-1α mRNA levels are also increased by exercise [10, 15, 16]. Higher intensity exercise causes a more dramatic increase in total PGC-1α mRNA levels, which may be due to an increase in promoter B transcription [18], although this study did not distinguish between isoforms. The PGC-1α response can be activated through treatment of cultured human muscle cells with AICAR and norepinephrine, suggesting a similar adrenergic activation pathway [15]. However, the molecular mechanisms of PGC-1α activation in response to these specific stressors are not yet understood.

One possibility for the increase of PGC-1α promoter B transcription during exercise is epigenetic regulation. The histone landscape is an important part of transcriptional activation [19, 20]. Specific histone modifications (e.g. H3K4me3) produce an open DNA conformation, allowing transcription factors access to gene promoters, whereas other histone modifications (e.g. H3K27me3) are frequently associated with silenced or repressed genes [20]. Furthermore, DNA methylation may also control transcriptional activity of genes, with methylated promoters transcribed at lower levels than unmethylated promoters [21–23].

We hypothesized that, under basal conditions in skeletal muscle, promoter A maintains an epigenetic landscape conducive to transcription (i.e. little DNA methylation, transcriptionally active histone marks), whereas promoter B is transcriptionally silenced. Furthermore, we hypothesized that the increase of PGC-1α mRNA from promoter B in skeletal muscle in response to acute exercise was due, in part, to changes in the epigenetic landscape of the DNA immediately surrounding the two promoters. To test this hypothesis, we examined the level of activating and repressing histone marks across each promoter. Additionally, we applied both bisulfite sequencing (BSFT-Seq) and Tet-assisted bisulfite sequencing (TAB-Seq) to determine whether DNA methylation or hydroxymethylation status, respectively, changed after exercise.

## Materials and Methods

### Animals

C57BL/KaLwRij female mice, aged about 3 months, were obtained from VCU’s transgenic core facility (VCU, Richmond VA, USA). Mice were caged in groups of four or fewer, and provided with food and water ad libitum in the vivarium. All animal experiments were performed according to the policies and guidelines of the Institutional Animal Care and Use Committee (IACUC) at Virginia Commonwealth University, USA. All procedures were reviewed and approved by the VCU IACUC prior to the conduct of this study. Animals were euthanized in the vivarium by using controlled release of CO_2_ from a cylinder into a chamber that allows good visibility of the animals and large enough to avoid crowding. All efforts were made to minimize the number of animals used and to minimize their suffering.

### Exercise Protocol

Mice were randomly assigned to sedentary control and exercise groups (n = 6 each group). Mice were initially trained to run on a 4-lane rotarod of 3.0 cm diameter (Columbus Instruments). Training was performed over 15 min, with the rotarod increasing in speed from 15–35 rpm over that timeframe. The mice were then rested 1 day before acute exercise. Acute exercise was carried out at speeds of 35–45 rpm in a one hour period. Mice were exercised at 35rpm for 20 min, 40 rpm for 30 min, and 45 rpm for 10 min. During each time point the mice were gently replaced on the rod whenever they fell. Both the sedentary control and the exercise group were euthanized with CO_2_ inhalation one hour post exercise, as described above, and skeletal muscle (quadriceps) was removed and snap frozen at -80°C.

### Absolute quantitation of mRNA levels by RT-qPCR

Approximately 30mg of tissue was used to extract RNA using miRNeasy kit (Qiagen). After checking RNA quality by capillary electrophoresis (Experion, BioRad), 1ug of RNA was reverse transcribed (iScript, BioRad) into cDNA using random hexamers. Primers for use in Geometric Mean Analysis were designed by Beacon Designer (S1 Table). qPCR was performed using a CFX96 (BioRad) system. For all muscle samples, mRNA levels of seven common reference genes (beta-actin, GAPDH, 18S rRNA, 14-3-3, topoisomerase 1, 36B4, cytochrome C1) were assayed using IQ Multiplex Powermix or SsoFast EvaGreen (BioRad) to select genes with least variability, based on GeNorm analysis in qbasePLUS (Biogazelle). GAPDH and beta-actin were the two least variable genes, and their geometric means were selected to normalize the relative level of PGC1α isoform mRNA in individual muscle samples (S1 Fig). Primers specific for PGC-1α-a, PGC-1α-b, PGC-1α-c, or total PGC-1α were used as previously described [8] (S1 Table). Isoform specific standards were generated from mouse brain cDNA using primers specific for PGC-1α-a, PGC-1α-b, or PGC-1α-c, with each amplicon containing common exons found in all three transcript species (S1 Table). These amplicons were inserted into the pCR-4TOPO vector (Life Technologies) and confirmed via sequence analysis. Absolute copy numbers were calculated using isoform specific plasmids as standards in the assays. Statistical analysis was performed using Student’s paired t-test.

### ChIP

ChIP was performed as previously described [24]. Skeletal muscle tissues ranging from 110–185 mg were minced with a sterile scalpel, placed in ~20 ml RPMI-1640 (Gibco) culture medium supplemented with 10% sterile fetal bovine serum and 1% formaldehyde (Ted Pella, Inc), and rocked for 10 minutes at 25°C. The fixation was quenched by adding glycine to a final concentration of 0.125M. Samples were centrifuged at 450 x g at 4°C for 10 minutes and the pellets were resuspended in 5ml cold PBS containing 1mM PMSF. The suspension was placed on ice and homogenized in a Dounce homogenizer then centrifuged at 450 x g at 4°C. The pellets were washed first with a buffer containing Triton (0.25%), EDTA pH8.0 (10mM), EGTA pH7.5 (0.5mM) and HEPES pH7.5 (10mM) containing 1ug/ml each pepstatin, leupeptin, aprotinin and 1mM PMSF. The pellets were again washed with a second buffer containing NaCl (0.2M), EDTA pH8.0 (1mM), EGTA pH7.5 (0.5mM) and HEPES pH7.5 (10mM) and 1ug/ml each pepstatin, leupeptin, aprotinin and 1mM PMSF and stored at -80°C. The lysis buffer used on muscle tissue contained 1% SDS as opposed to 0.1% SDS described previously. Rabbit-generated antibodies for H3K4me3 (Millipore, Cat# 07–473) or H3K27me3 (Millipore, Cat# 07–449) were used to immunoprecipitate histones. As a negative control, normal rabbit IgG (Cell Signaling Technology, Cat# 2729S) was used. Input samples were generated by taking the supernatant from a ChIP assay with no antibody. Samples were then subjected to qPCR using primers specific for 5 sites surrounding the transcriptional start sites of either Exon1a or Exon1b/c. qPCR was performed on a Chromo4 DNA-engine (BioRad) using SYBR Green (Qiagen). Statistical analysis was performed via ANOVA.

### Bisulfite Treatment of DNA and Amplicon Sequencing

DNA extracted from muscle (350ng/sample) was treated using the EZ DNA Methylation-Direct Kit (Zymo Research) according to the manufacturer’s instructions. DNA was eluted into 12ul of elution buffer. Primers were designed to generate multiple amplicons spanning all CpG dinucleotides within 1000bp upstream of the transcriptional start site. Each primer was fused to a M13F or M13R sequence for facilitation of sequencing. Primer sequences are shown in S1 Table. 1ul of bisulfite treated template was used to generate each amplicon. Amplicons were sequenced by both M13F and M13R primers to ensure full coverage. Sequence chromatograms were analyzed using Chromas Lite (Technelysium Pty Ltd). The peaks of both cytosine and thymine were measured at each CpG dinucleotide site, and a percentage of protected cytosines was calculated.

### Tet-assisted Bisulfite Sequencing

The 5hmC TAB-Seq Kit (WiseGene) was used to convert DNA extracted from mouse muscle from 3 of the 6 paired mice in both sedentary and exercised groups. The 5mC and 5hmC controls were generated as previously described [25]. 500ng of mouse muscle DNA was mixed with 2.5ng of the 5mC control and sheared using a bath sonicator (Bioruptor) for 5min (High setting, 30sec on/30sec off). DNA was then purified and concentrated using 2x volume of Ampure XP beads (Beckman-Coulter). 2.5ng of 5hmC control was added to the DNA mixture and all were treated with beta-glucosyltransferase for 1hr at 37°C. DNA was again purified and concentrated using 2x volume Ampure XP beads, followed by treatment with 25ug of the recombinant Tet1-catalytic domain for 1hr at 37°C. 25ug of Proteinase K was then added and the sample was incubated at 50°C for 1hr. DNA was again purified and concentrated using 2x volume of Ampure XP beads. The DNA was then bisulfite treated and sequenced as described in the preceding section. 5mC and 5hmC controls were amplified with primers described in S1 Table to control conversion of 5mC and protection of 5hmC. Statistical analysis was performed via ANOVA.

## Results

### Mouse exercise model

**Fig 1:**
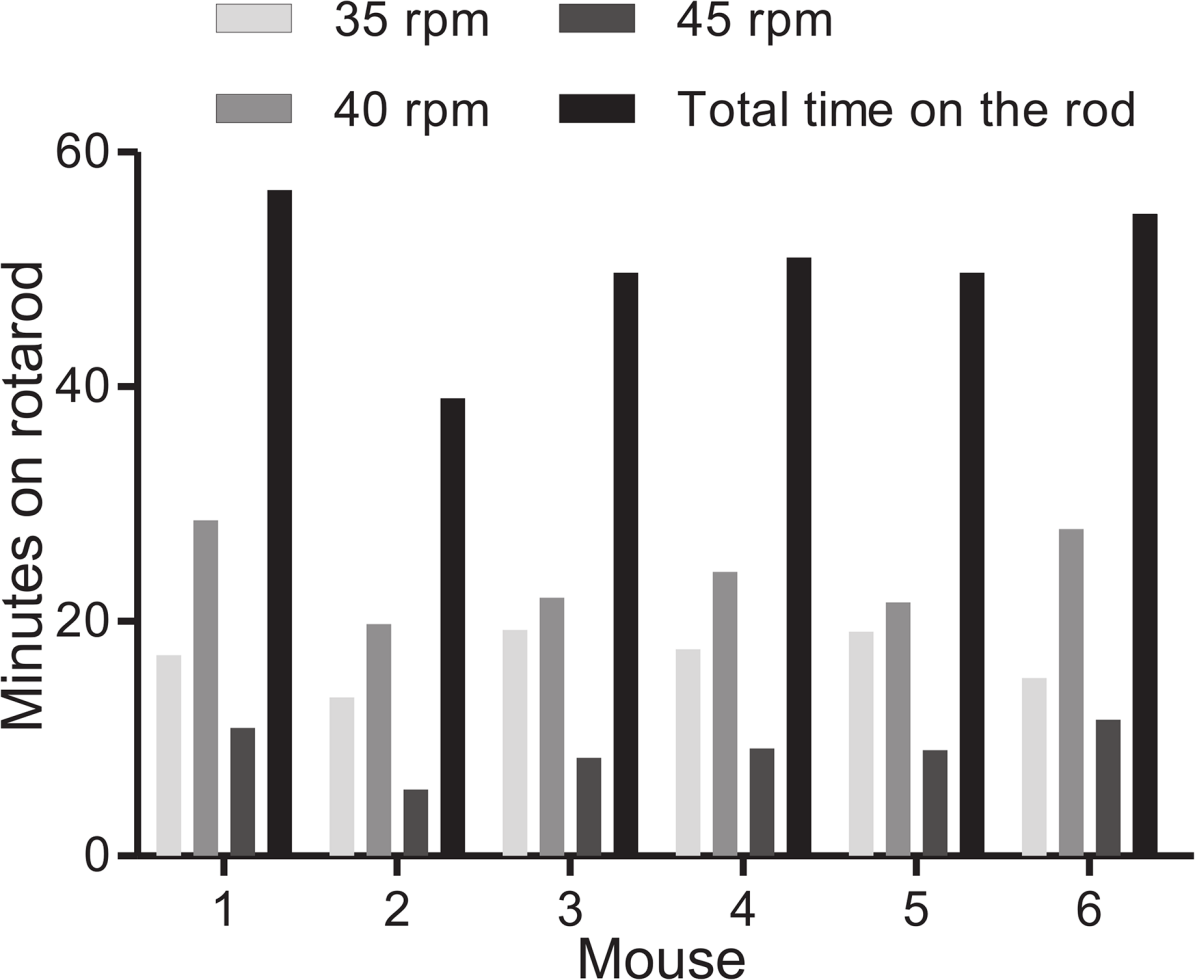
Mouse exercise session on a rotarod. After initial training, individual mice were run on an increasing rotarod speed of 35, 40, and 45 rpm. Graph shows time spent at each speed and cumulative time, in minutes, for each mouse.

Previous studies have clearly demonstrated the increase of PGC-1α after exercise [8, 9, 12]. Although previous studies were performed using a treadmill, we sought to replicate this induction using a rotarod. Six sets of mice were paired, with one remaining sedentary and the other exercised to exhaustion. Mice selected for exercise were first trained on the rotarod, which entailed a 15 minute bout of exercise on the rotarod with the speed increasing from 15 rpm to 35 rpm. The mice were rested for 1 day before being placed back on the rotarod for a round of acute, exhaustive exercise for a total of 1 hour. The mice were subjected to a speed of 35 rpm for 20 minutes, followed by 40 rpm for 30 minutes, followed by 45 rpm for 10 minutes. In the event that a mouse fell off the rotarod, it was gently replaced. The times each mouse spent at each speed are shown in Fig 1 and were comparable across the group of exercised mice.

### Levels of PGC-1α mRNA after exercise

**Fig 2:**
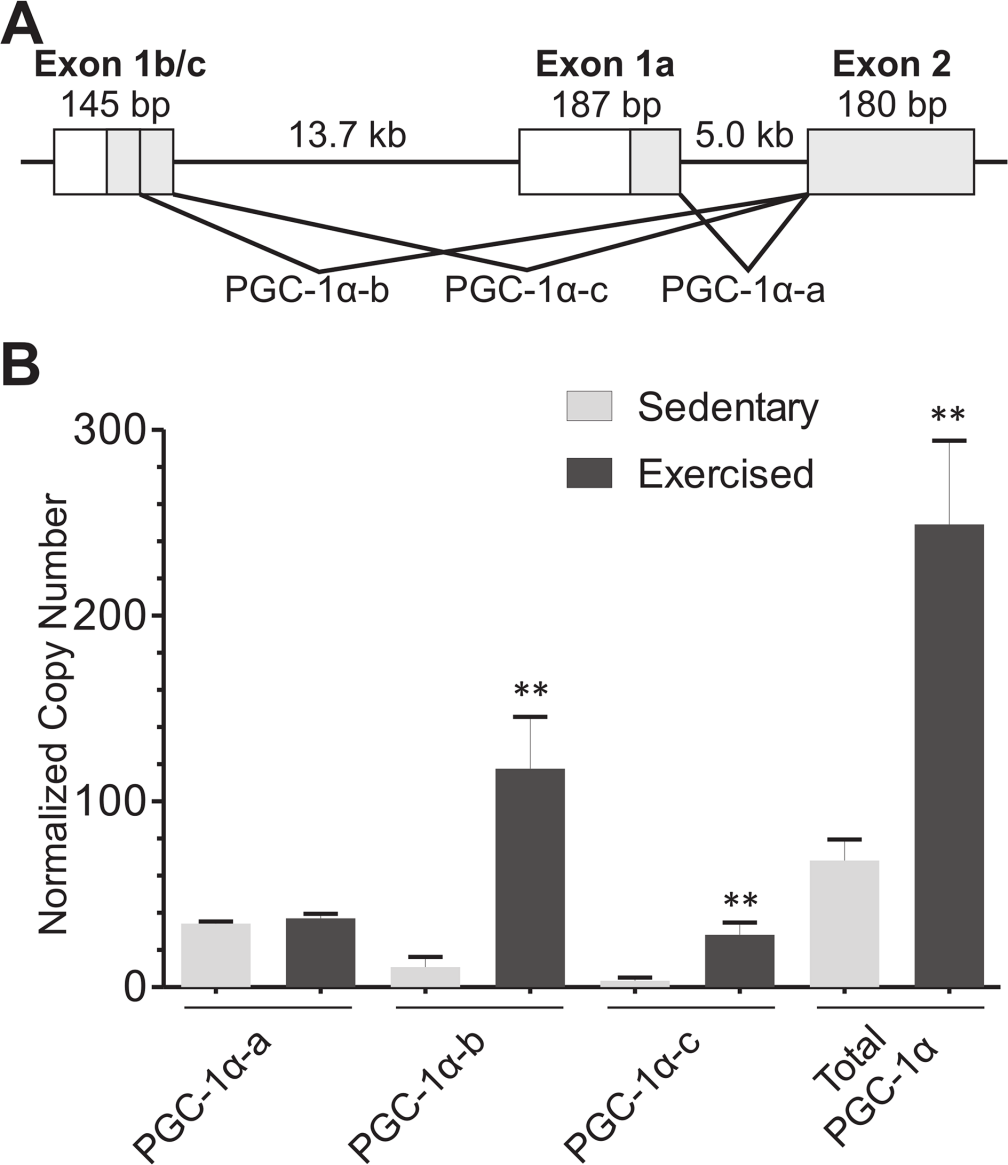
PGC-1α mRNA levels in skeletal muscle, by RT-qPCR, after rotarod exercise. (A) Schematic diagram of PGC-1α isoforms examined in this study. PGC-1α-a is expressed from one promoter, whereas PGC-1α-b and PGC-1α-c are both expressed from the same promoter. (B) Paired mice (n = 6 each group) were either exposed to rotarod exercise as described in Fig 1, or kept sedentary. RT-qPCR is shown for PGC1α using isoform-specific primers against PGC-1α-a, PGC-1α-b, or PGC-1α-c. Known quantities from separate cloned plasmids containing each isoform were used to quantitate absolute copy numbers of PGC-1α isoforms or total PGC-1α, which were normalized to geometric means of GAPDH and beta actin mRNA levels. Bars are shown ±SEM. (** p<0.005 by paired t-test).

At least four PGC1α mRNA species have been identified in mouse [10]. They are transcribed from two promoters, with two splice donors in Exon1b/c and one donor in Exon1a (Fig 2A). Splicing of PGC-1α-a, PGC-1α-b, or PGC-1α-c into the second exon should occur regardless of further 3’-splice variation. We asked whether the rotarod exercise model would induce transcription of PGC1α-b and PGC1α-c. We developed plasmids containing Exon 1a/Exon 2, Exon 1b/Exon2, or Exon 1b/c/Exon2 amplicons to serve as copy number controls, allowing the direct comparison of absolute amounts of mRNA present for PGC-1α-a, PGC-1α-b, or PGC-1α-c across the different primer sets. We therefore are measuring only the levels of mRNA transcribed from either promoter A or promoter B, including both possible splice variants from Exon 1b/c. However, these RT-qPCR experiments do not distinguish further 3’-splice variations that have been described in other studies [10, 14, 17].

We used RT-qPCR to quantitate the absolute levels of PGC-1α-a, PGC-1α-b, PGC-1α-c, or total PGC-1α mRNA in both sedentary and exercised mice (Fig 2B). A single bout of exhaustive exercise increased skeletal muscle total PGC-1α mRNA from a normalized copy number of 68.2 to 249.1, a 3.7-fold increase. As expected, PGC-1α-a did not significantly change (34.2 to 37.1). However, PGC-1α-b changed from 10.9 to 117.6, increasing by 10.8-fold. Similarly, PGC-1α-c increased from 3.4 to 28.2, an 8.3-fold increase. These data show that the rotarod exercise model is similar to that of the treadmill exercise. Previous studies indicated a discrepancy between the combined levels of PGC-1α-a, PGC-1α-b, and PGC-1α-c when compared to primers measuring total PGC-1α mRNA levels [8, 9], suggesting the presence of additional, as yet unidentified, promoters in skeletal muscle. However, using qPCR with an absolute quantitation method, we found that the sum of PGC-1α-a, PGC-1α-b, and PGC-1α-c is not significantly different from the quantity of total PGC-1α. Thus, the two promoters examined produce most or all of the PGC-1α transcripts in skeletal muscle.

### Histone modifications across PGC-1α promoters

**Fig 3:**
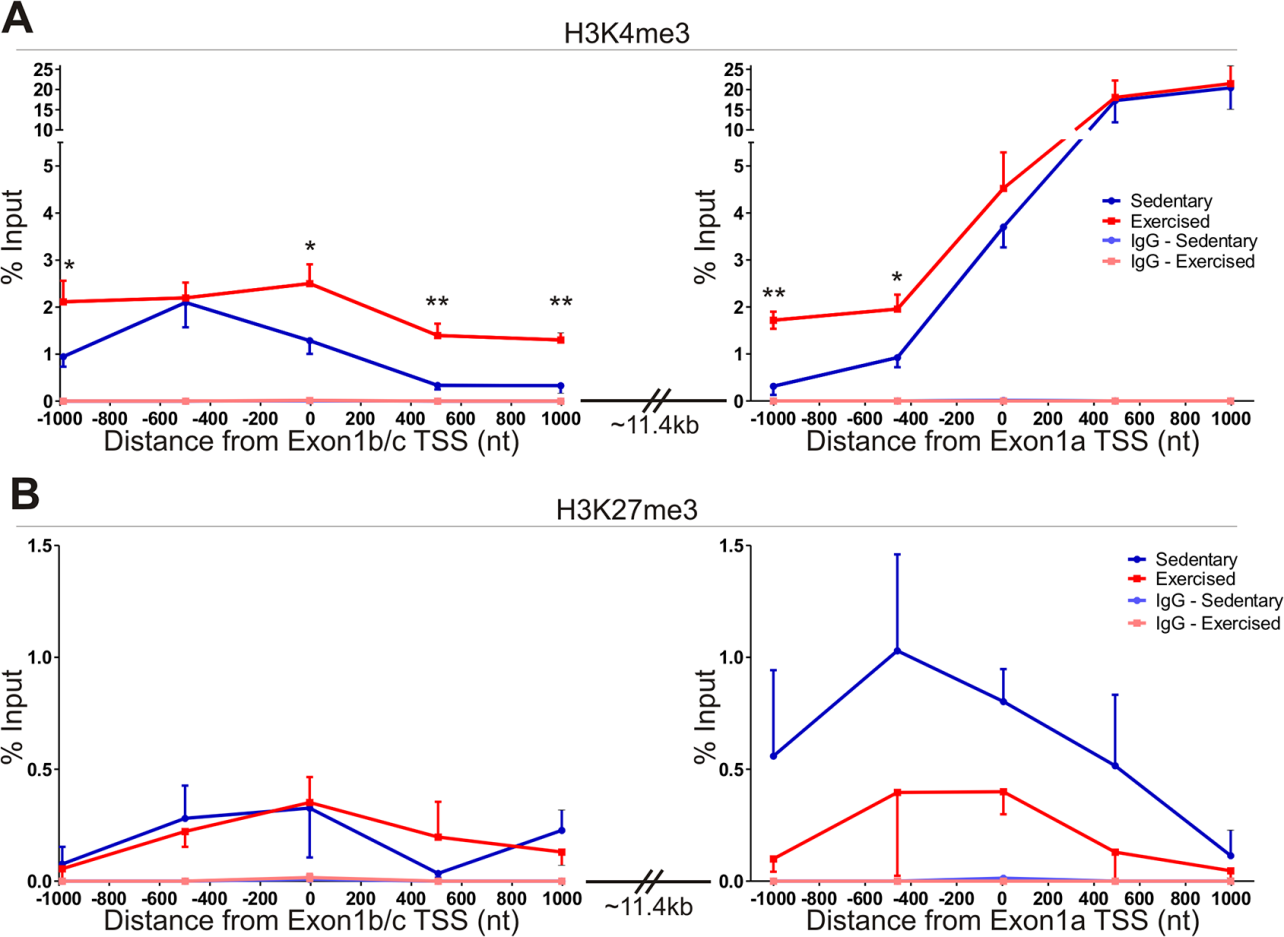
Chromatin Immunoprecipitation of histone modifications across Exon 1a and Exon 1b/c transcriptional start sites of PGC-1α. (A) Presence of an activating histone modification (H3K4me3) was examined via qPCR 1000bp in each direction from the transcriptional start site of Exon 1a (left) or Exon 1b/c (right). (B) Presence of a repressing histone modification (H3K27me3) was examined via qPCR 1000bp in each direction from the transcriptional start site of Exon 1a (left) or Exon 1b/c (right). 2-way ANOVA analysis found significant difference between the level of H3K4me3 only across the Exon 1b/c transcriptional start site (p<0.0001). Individual points were compared for statistical significance using student’s t-test (* p<0.05, ** p<0.005).

Specific histone modifications are observed at the promoters of transcriptionally active (H3K4me3, H3K27ac) or transcriptionally repressed (H3K27me3) genes [20, 26–28]. We asked whether, under basal conditions, histone marks indicating active transcription were present at the PGC-1α-a promoter, in contrast to an expected silenced PGC-1α-b/c promoter. Further, we tested whether the levels of epigenetic modifications changed during the activation of PGC-1α-b and PGC-1α-c during exercise. Since histone acetylation (e.g. H3K27ac) appears to be required for CpG dense promoters [20], whereas the H3K4me3 histone mark is associated with actively transcribed CpG sparse promoters [20, 27], we therefore hypothesized that if changes in H3K4me3 are important for activation of isoform-specific PGC1a transcription, we would observe an increase across promoter B, whereas promoter A should remain unchanged. To test this hypothesis, we performed chromatin immunoprecipitation (ChIP) on formaldehyde cross-linked muscle samples from the same individual mice used for mRNA quantitation. In general, the activating H3K4me3 mark was 10-fold lower across promoter B than promoter A, while the H3K27me3 repressive mark was low over both promoters. The basal level of H3K4me3 across a 1kb region prior to the transcriptional start site of Exon 1a (Fig 3A, right panel, blue line) was similar to the corresponding 1kb region prior to the transcriptional start site of Exon 1b/c (Fig 3A, left panel, blue line). However, at and beyond the transcriptional start site, the amount of H3K4me3 detected at Exon 1a was 3- to 62-fold higher than the same region past the Exon 1b/c transcriptional start site, in accord with the observation that the basal level of PGC-1α-a mRNA is approximately 3-fold higher than the combined level of PGC-1α-b and PGC-1α-c mRNAs in skeletal muscle under basal conditions. Following exercise, there is an overall increase (between 2- and 4-fold) in the 2kb region across promoter B (Fig 3A, left panel, red line). This difference was found to be significant by 2-way ANOVA. There is also a significant increase in the level of H3K4me3 at two points before the transcriptional start site of Exon 1a (5-fold and 2-fold increase, respectively; Fig 3A, right panel, red line). These changes were detected comparing individual points via student’s t-test. These data suggest that the DNA near and after the transcriptional start site of Exon 1b/c is in a more open conformation following exercise. Further, it indicates that the DNA approaching the transcriptional start site of Exon 1a, which Pol II must traverse during transcription of PGC-1α-b and PGC-1α-c mRNA, is also in an open conformation. Together, these data help to explain how PGC-1α-b and PGC-1α-c mRNA are transcriptionally upregulated during exercise.

The level of the repressing histone mark, H3K27me3, across both promoter A and promoter B (Fig 3B) was very low, with little difference between promoter A and promoter B in sedentary mice, and no significant change between sedentary and exercised mice across either promoter. Thus, we conclude that the repressing histone mark, H3K27me3, does not play a role in regulating isoform-specific PGC1a transcription during exercise.

### Bisulfite sequencing analysis of PGC-1α after exercise

Because we observed a change in the activating histone modification, H3K4me3 present around promoter B following exercise, we asked whether there might be a concomitant change in DNA modifications in either promoter A or promoter B. The presence of methylation at CpG dinucleotides is a hallmark of repressed transcription [22]. Because PGC-1α-a mRNA levels were higher than PGC-1α-b and PGC-1α-c mRNA under basal conditions, we predicted higher CpG methylation across promoter B compared to promoter A in sedentary mice. However, because CpG methylation is associated with long-term changes [29], we did not expect to see a change in PGC-1α promoter methylation after stimulation with exercise.

**Fig 4:**
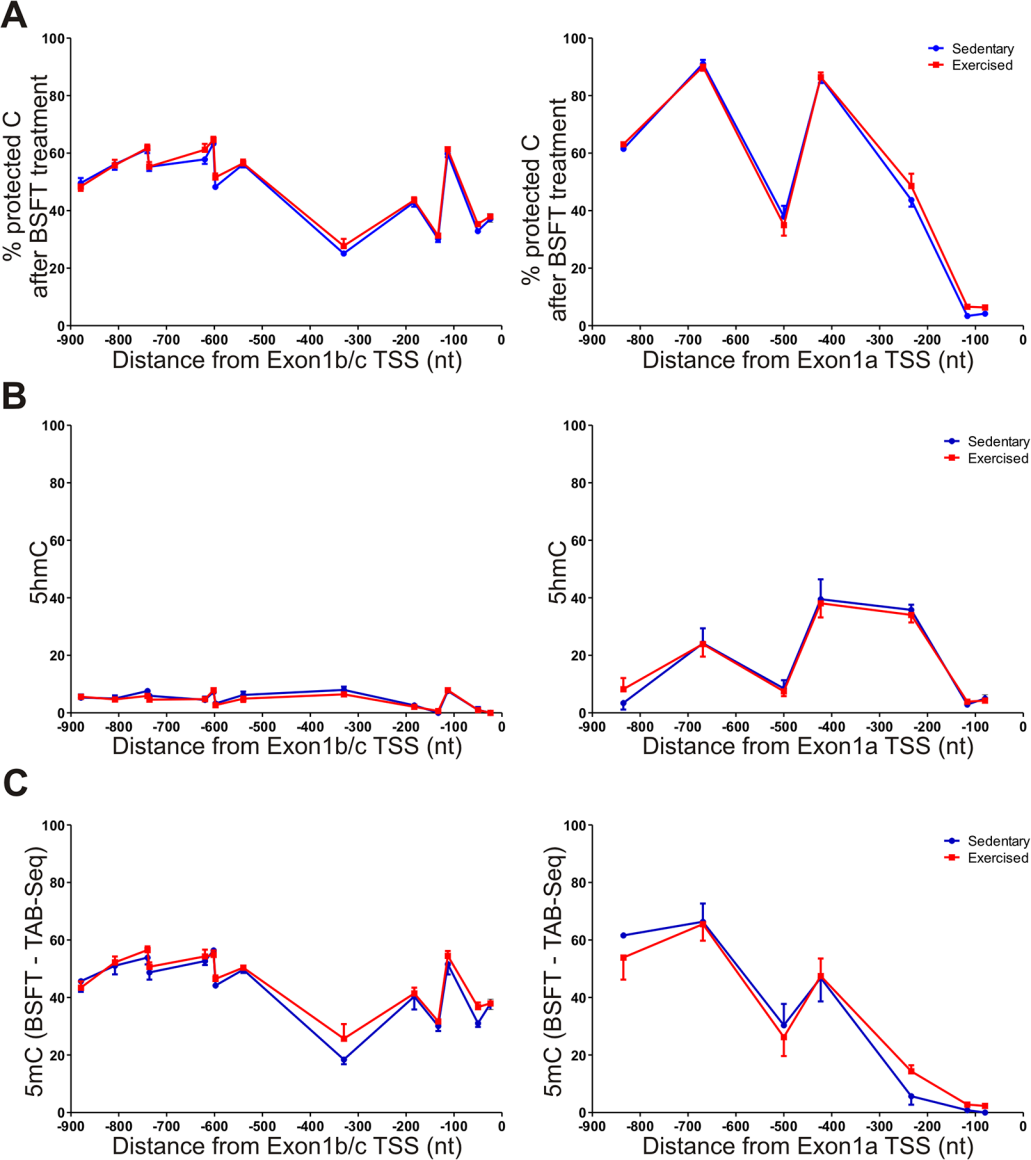
Measurement of 5mC and 5hmC across PGC-1α Exon1a and Exon1b/c promoter regions. (A) Percent protected cytosines at each CpG site within the promoter region of PGC-1α Exon1a (right) or Exon1b/c (left) after bisulfite treatment of skeletal muscle DNA. (B) Percent protected cytosines after TAB-Seq, showing the level of 5hmC at each CpG site. (C) The level of 5mC is shown at each CpG site across the PGC-1α promoters.

We first applied BSFT-Seq using skeletal muscle genomic DNA extracted from the same individual mice as used for the RT-qPCR and ChIP assays. Following bisulfite modification [30], we PCR-amplified overlapping fragments encompassing a 1kb region upstream of the transcriptional start sites of either Exon 1a or Exon 1b/c and covering all CpG dinucleotides in these CpG sparse promoters. The 5’ ends of the amplification primers carried the M13F and M13R priming sites to facilitate direct sequencing. Amplicon sequencing has previously been used for bisulfite sequencing analysis [31], and allows the detection of protected cytosines at a cellular population level. In unexercised mice, both promoters contained a high percentage of protected cytosines (Fig 4A). Surprisingly, promoter A generally had a higher level of protected cytosines within the 900bp region examined, ranging from 38% to 90% (Fig 4A, right panel). In contrast, the 900bp region of promoter B ranged from 25% to 65% (Fig 4A, left panel). However, promoter A had little cytosine protection within 150bp of the transcriptional start site (3–4%), whereas promoter B retained a substantial level of cytosine protection within 200bp of the transcriptional start site (33–37%). Thus, DNA methylation likely maintains the low basal mRNA level from promoter B.

Given that CpG methylation is a hallmark of transcriptional repression, our data correlate with the basal mRNA levels from each promoter, in that there is little DNA modification close to the transcriptional start site of Exon 1a, and substantial modification near the start of Exon 1b/c. Furthermore, there was no notable change in protected cytosines following a single bout of exercise, demonstrating that PGC-1α promoter methylation status remains unchanged after acute exercise.

### 5mC and 5hmC levels at the PGC-1α promoters

One major caveat of bisulfite treatment is the inability to differentiate between methylated (5mC) and hydroxymethylated (5hmC) cytosines. This distinction is important as 5mC is involved in transcriptional repression [21–23], while 5hmC plays a role in demethylation and/or transcriptional activation [32, 33]. It was therefore possible that, although the level of cytosines protected from bisulfite treatment did not change following acute exercise, the ratio of 5mC/5hmC changed, which would indicate a transition from repressed to activated transcription. We therefore determined the levels of both 5mC and 5hmC across the promoter A and promoter B, using TAB-Seq, which leaves only 5hmC protected from bisulfite modification [25, 34]. In this assay, 5hmC nucleotides are first protected by glucosylation through β-glucosyltransferase, forming 5Glc-hmC. Recombinant Tet1 dioxygenase converts all 5mC through 5hmC to 5-carboxylcytosine (5caC) and 5-formylcytosine (5fC), which are no longer protected from bisulfite modification. Thus, during subsequent bisulfite treatment of the DNA, only the 5Glc-hmC is protected and read as cytosine, whereas all other modified and unmodified cytosines are converted to uracil. The level of 5hmC from TAB-Seq is subtracted from the level of total protected cytosines from BSFT-Seq to obtain the true 5mC level.

The DNA from three pairs of mice (exercised or sedentary), from the same muscle samples used in the BSFT-Seq, was subjected to TAB-Seq. Control DNA fragments containing either 5mC or 5hmC were added to each sample prior to treatment [25] to determine the degree of protection (5hmC) and Tet1 conversion (5mC) in each treatment. These controls showed >85% 5hmC protection and >95% 5mC conversion (S2 Fig), comparable to that obtained by Yu et. al. [25]. The same primers used in the BSFT-Seq assay were used to generate amplicons for direct sequencing. Data was analyzed at promoter A and promoter B, and plotted as percent 5hmC (Fig 4B, right and left panels, respectively). There was practically no 5hmC present within promoter B, with no CpG site above 8% protected cytosines. This result indicates that most or all of the protected cytosines detected during BSFT-Seq were 5mC (Fig 4C, left panel), and the levels did not change with exercise.

Interestingly, there was a substantial amount of 5hmC within promoter A, ranging from 3–40% (Fig 4B, right panel). Most of the 5hmC was found within 400bp from the transcriptional start site of Exon 1a. The 5mC levels (Fig 4C, right panel), are therefore substantially lower than that obtained by BSFT-Seq. Because 5hmC is found in transcriptionally active genes [33], it is likely that the lack of cytosine methylation and the presence of 5hmC close to the transcriptional start site provides epigenetic explanation for the higher basal mRNA level of PGC-1α-a when compared to that of PGC-1α-b and PGC-1α-c, and suggests a role for 5hmC in maintaining active, CpG sparse promoters in an open configuration for transcriptional activation.

## Discussion

Previous studies have demonstrated that exercise increases the level of PGC-1α-b and PGC-1α-c mRNA in the skeletal muscle of mice, while leaving the amount of PGC-1α-a relatively unchanged [8, 18]. We found that exercise using a rotarod produced a large increase in the mRNA levels of PGC-1α-b and PGC-1α-c, similar to that seen in previous treadmill exercise experiments [18]. These previous experiments revealed a disparity between total PGC-1α and the sum of PGC-1α-a, PGC-1α-b, and PGC-1α-c [8, 9, 18], with the total level of PGC-1α mRNA higher than the sum of the individual isoforms. This suggested additional, as yet unidentified promoters and isoforms. However, our studies using normalized copy numbers for each mRNA show that the sum of each individual mRNA is similar to the total amount of PGC-1α mRNA. Thus, these three isoforms comprise most or all of the mRNA present within skeletal muscle.

In this work we sought to determine whether epigenetic regulation was involved in the selective activation of PGC-1α-b and PGC-1α-c following acute exercise. Methylation at CpG islands is thought to control long-term, stable repression of genes [35]. CpG islands are described as having a GC content of at least 55% and a CpG observed-to-expected ratio of 0.65 within a 500 bp region [36]. Neither promoter A nor promoter B has a CpG island within the regions examined. Some studies examining non-CpG island promoters have shown CpG methylation in tissues not expressing these genes, and lack of methylation in expressing cells [37, 38]. However, studies on genome-wide methylation-expression relationships have yielded mixed results, leaving little evidence either way for the role of methylation in non-CpG island promoters [39, 40].

We observed CpG methylation present on both PGC-1α promoters. Near the transcriptional start site, the level of methylation was higher on promoter B (~40%) than on promoter A (<10%). Interestingly, although we found multiple CpG sites with a higher percentage of protected cytosines on promoter A, most of these cytosines are modified with a hydroxymethyl group. Thus, the DNA within 250nt of the transcriptional start site of promoter A has very little CpG methylation (<10%). This observation underscores the importance of differentiating 5mC from 5hmC, which is not possible using bisulfite treatment alone. Our data also supports an active role for 5hmC in maintaining an open chromatin configuration around such CpG sparse active promoters [33].

We found a correlation between the level of methylation and PGC-1α isoform mRNA levels. Promoter A, with relatively little methylation was expressed 2.4-fold higher than Promoter B (combined PGC-1α-b and PGC-1α-c levels). These data suggest that methylation of non-CpG island promoters might be involved in fine-tuning the basal level of mRNA within a tissue. Tissue specific comparisons of mRNA levels and DNA methylation will be needed to test this possibility. Furthermore, altering the level of methylation on promoter A and B may give further insight into whether methylation plays a functional or correlative role in basal mRNA levels. Our work also clearly shows no significant methylation change in either promoter following exercise. The lack of exercise-associated change indicates that while DNA methylation may be involved in controlling basal mRNA levels, it is not involved in the activation of promoter B, nor does it prevent this activation from occurring. This finding is not surprising given that methylation is associated with long-term transcriptional control [29], whereas our experiments looked at a short, acute bout of exercise. The possibility that changes in DNA methylation at either promoter occur during a prolonged period of exercise, for weeks or months, remains to be investigated. It is also possible that the change in methylation occurs within a smaller time-frame than we examined. Using methylated DNA immunoprecipitation (MeDIP), a previous study observed decreasing methylation at the PGC-1α promoter immediately after cessation of exercise, whereas the dramatic increase in PGC-1α mRNA occurred 3-hours post-exercise [41]. We looked 1-hour post exercise, which may be too late to see any methylation changes. However, given that Barres, et al only looked at promoter A methylation with respect to total PGC-1α mRNA levels, it is difficult to compare activation of promoter B and the methylation data presented there. Additionally, Barres et al have shown non-CpG methylation near promoter A [41, 42]. We did not see non-CpG methylation in our studies, which may be due to either a species difference (human vs. mouse) or an assay difference (clonal sequencing vs. amplicon sequencing).

Most studies examining the role of histone modifications and activation of genes are correlative in nature. It is technically difficult to specifically alter the histone status of a single promoter. Global alterations to histone modifications may lead to complications for an individual [43], and would likely complicate any analysis of a single gene which relies on many other proteins for transcriptional activation. In our studies, we found that acute exercise-induced increase of PGC-1α-b and PGC-1α-c mRNA coincided with an increase in the activating histone mark, H3K4me3, across the transcriptional start site of Exon 1b/c. In contrast, neither PGC-1α-a mRNA levels nor H3K4me3 residency after the transcriptional start site of Exon 1a were changed after exercise. However, H3K4me3 levels increased prior to the transcriptional start site of Exon 1a, similar to what was observed at Exon 1b/c. The histone mark H3K4me3 is indicative of transcriptional activity on non-CpG island promoters [20, 27]. H3K4me3 has also been shown to be a direct indicator of RNA Pol II elongation in both yeast and higher eukaryotes [44, 45]. The increased transcription of PGC-1α-b and PGC-1α-c mRNA following acute exercise may be due to a relaxation of the DNA in the presence of elevated H3K4me3 [20], allowing transcription factors access to the promoter. One possibility for a tissue specific transcription factor would be MyoD, which has been shown to bind to DNA sequences within promoter B in *in vitro* studies [13]. Alternatively, it has been suggested that if the H3K4me3 histone mark is not responsible for the direct transition of RNA Pol II from a paused to elongating state, H3K4me3 instead serves as an indicator that the switch to Pol II elongation has occurred [46]. In either scenario, these data suggest that the increase in H3K4me3 we observed after exercise in mouse skeletal muscle is indicative of increased PGC-1α-b/c transcription due to an increase in Pol II elongation.

A second possibility for the rapid activation of PGC-1α-b and PGC-1α-c is the engagement of an enhancer. Several genes have now been identified as having “poised promoters,” allowing for rapid activation of mRNA transcription in response to specific signals [47]. In vertebrates, many genes critical for developmental pathways exist as pre-existing complexes (for example *Hox* and *Shh* loci), in which PolII is held in place between the gene promoter and a distant stretch of enhancer DNA by transcription factors, chromatin modifying factors (i.e. p300 or SWI/SNF), and Cohesins [48, 49]. This DNA-protein complex remains poised until tissue specific transcription factors release PolII from its arrest, allowing rapid initiation of transcription. At other loci, long range interactions between enhancers and promoters occur *de novo*, triggered by specific transcription factors [49]. Low to moderate levels of H3K4me3 and H3K27me3 frequently colocalize with both types of promoters [27, 50–52]. The presence of both H3K4me3 and H3K27me3 across promoter A and promoter B suggests that PGC-1α may utilize poised promoters for isoform-specific and tissue-specific transcription activation. In liver PGC-1α-a is specifically increased upon fasting, and brown adipose tissue shows an increase of PGC-1α-b and PGC-1α-c on exposure to cold, but increased PGC-1α-a in response to β-adrenergic agonists [8], suggesting that if one or more enhancers are present, they may be both tissue- and stress-specific. Therefore, tissue-specific studies will be needed to determine the molecular mechanisms responsible for activation of either promoter A or promoter B, and whether the epigenetic profiles described here are predictive of tissue- and stress-specific enhancers.

## Supporting Information

S1 ARRIVE Checklist ARRIVE Checklist. Checklist required for *in vivo* animal studies.
(PDF)

S1 Fig Geometric mean analysis of exercised and sedentary mice. Stable gene targets across treatment conditions will generate lower geNorm M values. The Beta Actin and GAPDH targets were selected as the least variable between sedentary and exercised mice.
(TIF)

S2 Fig TAB-Seq control amplicon analysis. (A) The first 8 CpG dinucleotides of the 5mC control plasmid are plotted as an average for % conversion rate. (B) The first 9 cytosine residues after the start of sequencing are plotted as an average for % protection.
(TIF)

S1 Table List of all primers and probes used in this study. All primers are listed in a 5’ to 3’ orientation.
(XLSX)
