# Supplementary figures and images for: Epigenetic Modifications of the PGC-1α Promoter during Exercise Induced Expression in Mice
Source: PLoS One. 2015 Jun 8;10(6):e0129647. doi: 10.1371/journal.pone.0129647 (PMC4460005; doi:10.1371/journal.pone.0129647)

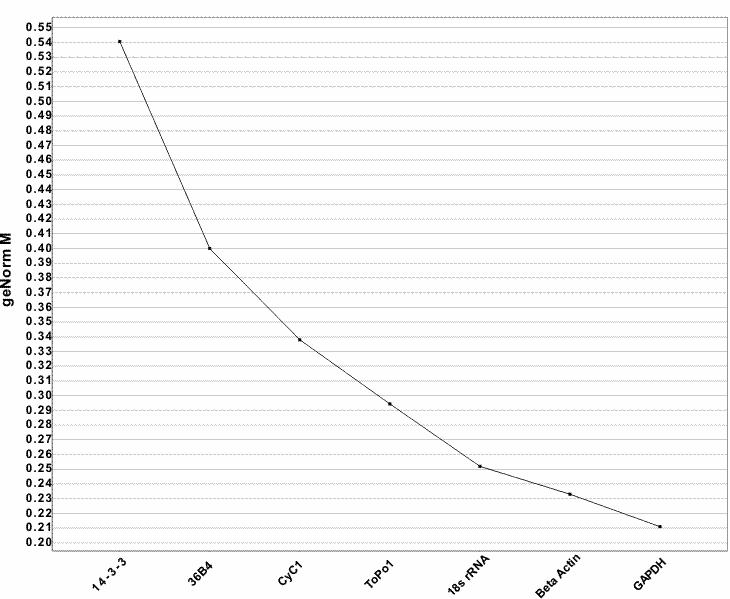

Supplement: S1 Fig — Stable gene targets across treatment conditions will generate lower geNorm M values. The Beta Actin and GAPDH targets were selected as the least variable between sedentary and exercised mice. (TIF) [file pone.0129647.s002.tif]

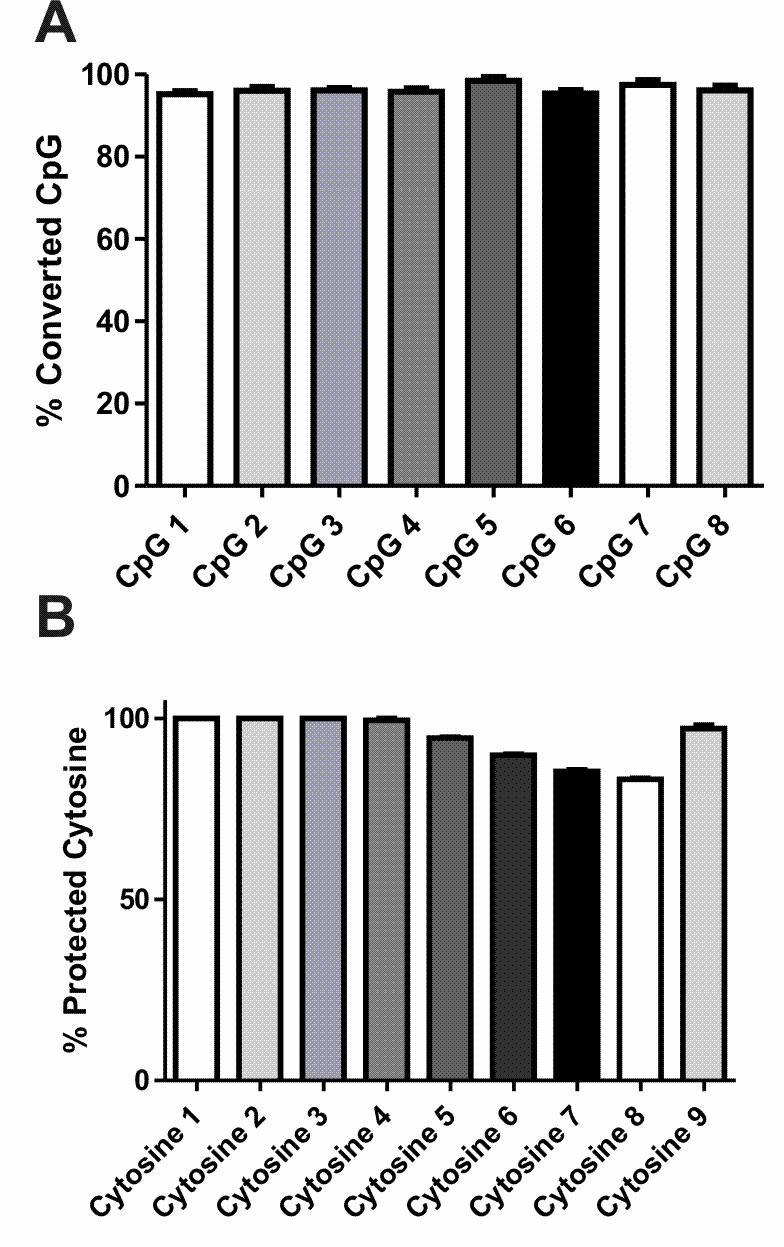

Supplement: S2 Fig — (A) The first 8 CpG dinucleotides of the 5mC control plasmid are plotted as an average for % conversion rate. (B) The first 9 cytosine residues after the start of sequencing are plotted as an average for % protection. (TIF) [file pone.0129647.s003.tif]
